# Actinobacteria Associated With Arbuscular Mycorrhizal *Funneliformis mosseae* Spores, Taxonomic Characterization and Their Beneficial Traits to Plants: Evidence Obtained From Mung Bean (*Vigna radiata*) and Thai Jasmine Rice (*Oryza sativa*)

**DOI:** 10.3389/fmicb.2018.01247

**Published:** 2018-06-11

**Authors:** Krisana Lasudee, Shinji Tokuyama, Saisamorn Lumyong, Wasu Pathom-aree

**Affiliations:** ^1^Department of Biology, Faculty of Science, Chiang Mai University, Chiang Mai, Thailand; ^2^Department of Applied Biological Chemistry, Faculty of Agriculture, Shizuoka University, Shizuoka, Japan; ^3^Center of Excellence in Bioresources for Agriculture, Industry and Medicine, Faculty of Science, Chiang Mai University, Chiang Mai, Thailand

**Keywords:** actinobacteria, arbuscular mycorrhizal spore, *Funneliformis mosseae*, plant growth-promoting activity, indole-3-acetic acid (IAA), rice (*Oryza sativa*), drought stress, low nutritional soil

## Abstract

In this study, we report on the isolation of actinobacteria obtained from spores of *Funneliformis mosseae* and provide evidence for their potential in agricultural uses as plant growth promoters *in vitro* and *in vivo*. Actinobacteria were isolated from spores of *F. mosseae* using the dilution plate technique and media designed for the selective isolation of members of specific actinobacterial taxa. Six strains namely 48, S1, S3, S4, S4-1 and SP, were isolated and identified based on16S rRNA gene sequences. Phylogenetic analysis showed that isolate SP belonged to the genus *Pseudonocardia* with *P. nantongensis* KLBMP 1282^T^ as its closest neighbor. The remaining isolates belonged to the genus *Streptomyces*. Two isolates, 48 and S3 were most closely related to *S. thermocarboxydus* DSM 44293^T^. Isolates S4 and S4-1 shared the highest 16S RNA gene similarity with *S. pilosus* NBRC 127772^T^. Isolate S1 showed its closest relationship with the type strain of *S. spinoverrucosus* NBRC14228^T^. The ability of these isolates to produce indole-3-acetic acid (IAA), siderophores and the ability to solubilize phosphate *in vitro* were examined. All isolates produced siderophores, four isolates produced IAA and two isolates solubilized inorganic phosphate at varying levels. *S. thermocarboxydus* isolate S3 showed the highest IAA production with high activities of phosphate solubilization and siderophore production. The inoculation of mung beans (*Vigna radiata*) with this strain resulted in a significant increase in fresh weight, root length and total length as an effect of IAA production. In an experiment with rice (*Oryza sativa*), *S. thermocarboxydus* isolate S3 promoted the growth of rice plants grown in low nutritional soil under induced drought stress. This report supports the view that the inoculation of rice with plant growth promoting actinobacteria mitigates some adverse effects of low nutrient and drought stress on rice.

## Introduction

Actinobacteria are Gram-positive mostly filamentous bacteria with high %G+C content in their genomes. They are prolific producers of useful bioactive metabolites with a wide spectrum of uses in particular antibiotics (Bérdy, 2005; Kurtboke, 2012; Tiwari and Gupta, 2012). It is also well accepted that some actinobacteria are considered potential plant growth promoting bacteria (Doumbou et al., 2001; Hameda and Mohammadipanah, 2015; Qin et al., 2017). Their ability to produce phytohormones (IAA), siderophores and to solubilize inorganic phosphates has been well-documented (Manulis et al., 1994; Rungin et al., 2012; Sadeghi et al., 2012; Lin and Xu, 2013; Anwar et al., 2016; Passari et al., 2016). The growth promotion of several plants by actinobacteria was reported in peas (Tokala et al., 2002), beans (Nassar et al., 2003), alfalfa (Le et al., 2016), wheat (Sadeghi et al., 2012; Anwar et al., 2016), and rice (Rungin et al., 2012; Gopalakrishnan et al., 2014).

Arbuscular mycorrhizal fungi (AMF) are well-known plant growth promoters and have been used in the agricultural industry worldwide (Smith and Smith, 2011). AMF can only be grown with plants as obligate symbionts (Owen et al., 2015). This symbiosis improves the plant’s nutrient uptake and provides AM fungi with carbon sources. *Funneliformis* is a member of AMF in the family *Glomeraceae*, which form symbiotic relationships with plant roots. It was formerly known as *Glomus mosseae* until the genus *Funneliformis* was established in 2010 with *F. mosseae* as a type species (Schubler and Walker, 2010). *F. mosseae* is widely used in horticulture as a bioinoculant (Kruger et al., 2012). This symbiosis improves the plant’s nutrient uptake and provides AM fungi with carbon sources. Recently, microorganisms associated with AM fungal spores have been reported, including several bacterial species that are both Gram-negative (Xavier and Germida, 2003; Bharadwaj et al., 2008a,b; Battini et al., 2016; Lasudee et al., 2017) and Gram-positive bacteria including actinobacteria of the genus *Streptomyces* (Schrey et al., 2012; Mohandas et al., 2013; Poovarasan et al., 2013; Battini et al., 2016). These mycorrhizal associated bacteria have shown interesting properties such as those associated with biocontrol and plant growth promoting activities (Mohandas et al., 2013; Poovarasan et al., 2013; Battini et al., 2016). The beneficial properties of these bacteria are considered attractive for sustainable agricultural practices. However, few research studies have been published on actinobacteria that are associated with mycorrhizal spores. This has led to our interest in mycorrhizal-associated actinobacteria, which may be a good source of novel taxa for the purposes of agricultural bioprospecting. Hence, it is the aim of this study to isolate actinobacteria from *F. mosseae* spores and to screen for their plant growth promoting properties both *in vitro* and *in planta* using mung beans (*Vigna radiata*) and Thai jasmine rice (*Oryza sativa*). We have given special attention to testing the ability of these actinobacteria to promote rice growth under drought conditions in soil possessing low nutritional content. Their taxonomic positions were also identified.

## Materials and Methods

### Selective Isolation of Actinobacteria From *Funneliformis mosseae* Spores

*Funneliformis mosseae* CMU-RYA08 was used for the isolation of actinobacteria in this study. It was originally isolated from the soil of a *Aquilaria crassna* plantation in Rayong Province, Thailand (Chaiyasen et al., 2014) and was kindly supplied to us by Dr. Amornrat Chaiyasen, Department of Biology, Faculty of Science, Chiang Mai University. Briefly, spores of *F. mosseae* were surface sterilized by vortex mixing them sequentially in 500 μl of 2% (v/v) sodium hypochlorite solution for 1 min, followed by 500 μl of 70% (v/v) ethanol for 1 min. After that, spores were washed with 500 μl of distilled water for 1 min (three times) and used for isolation of actinobacteria. The efficiency ofsurface sterilization method was determined by spreading the final washing water onto the nutrient agar as well as the International *Streptomyces* Project (ISP) medium 2 agar (Shirling and Gottlieb, 1966), and the plates were incubated at 30°C for 48 h. Twenty surface sterilized spores of *F. mosseae* were used for each isolation. Aseptically, 20 surface sterilized spores were added to microcentrifuge tubes containing 200 μl of soil extract broth and were ground by sterile micropestle. Soil extract was prepared according to the method of Taylor and Lochhead (1938). One kilogram of garden soil was sterilized in 1 l of tap water for 20 min at 121°C. The suspension was allowed to settle and the supernatant was filtered through Whatman’s No. 41 filter paper. One gram of glucose was added to the filtrate and the pH value was adjusted to 6.8–7.0 prior to being autoclaved. The resultant solution was used as a soil extract broth for enrichment. The resulting spore suspension was enriched by shaking at 120 rpm for 1 h at room temperature. Two hundred microliters of the sample were spread on starch casein agar (Küster and Williams, 1964) and humic acid-vitamin agar (Hayakawa and Nonomura, 1987) supplemented with 25 μl/ml nalidixic acid and 100 μg/ml ketoconazole. Plates were then incubated at 30°C for up to 4 weeks. All isolates were routinely cultivated on International *Streptomyces* Project (ISP) medium 2 agar (Shirling and Gottlieb, 1966) at 30°C and spore suspension was maintained in 20% (w/v) glycerol at -20°C for long term preservation.

### Molecular Identification by 16S rRNA Gene Sequencing

Biomass for molecular study was prepared by growing the isolates on ISP medium 2 agar for 7 days. Genomic DNA was extracted using FavorPrep Tissue Genomic DNA Extraction Mini Kit (Favogen) according to the manufacturer’s protocol. All isolates were subjected to amplification of 16S rRNA gene using universal primer 27F (5′-AGAGTTTGATCMTGGCTCAG-3′) and 1525R (5′-AAGGAGGTGWTCCARCC-3′) (Lane, 1991). PCR was carried out in a GeneAmp PCR System 9700 with the following reactions. The initial denaturation step was done at 94°C for 2 min, followed by 30 cycles of 94°C for 30 s, 55°C for 30 s and 72°C for 30 s and final extensions at 72°C for 7 min. PCR products of expected size were confirmed by 1% (w/v) agarose gel electrophoresis. The purified PCR products were sequenced commercially at 1st BASE DNA Sequencing Division, Malaysia. The obtained sequences were compared with related sequences in the EzBioCloud database^1^ using BLAST program. Phylogenetic analysis based on a neighbor-joining method (Saitou and Nei, 1987) was carried out using MEGA version 5.2 program (Tamura et al., 2011). The resultant tree topology was evaluated by bootstrap analysis (Felsenstein, 1985) of the neighbor-joining data based on 1000 re-sampled datasets

### *In Vitro* Plant Growth Promoting Potential

#### Indole-3-Acetic Acid (IAA) Production

The production of IAA of all isolates was determined by the colorimetric method as has been described previously (Khamna et al., 2010). Agar plug (5 mm diameter) of actinobacteria grown on ISP medium 2 agar was inoculated into 5 ml of ISP medium 2 broth containing 2 mg ml^-1^ of L-tryptophan (Gordon and Weber, 1951). Tubes were incubated at 28 ± 0.2°C with shaking at 110 rpm for 7 days. The supernatant was collected by centrifugation at 11,000 rpm for 5 min. IAA production was detected by mixing one ml of the supernatant with 2 ml of Salkowski’s reagent (Glickmann and Dessaux, 1994). The appearance of a pink color after incubation at room temperature for 30 min in the dark was an indicator of IAA production. The optical density was measured at 530 nm using a spectrophotometer (GENESYS TM 20 Visible Spectrophometer, Thermo Fishcer Scientific). The IAA concentration in the culture broth was estimated based on a calibration curve of pure IAA standard. The confirmation of IAA production was also confirmed by TLC analysis with 10 mg/ml IAA standard (Mohite, 2013). In addition, the *iaaM*, the key IAA biosynthetic gene of the highest IAA producing isolate, was examined by PCR amplification (Lin and Xu, 2013). The amplified gene (1,698 bp) was cloned and sequenced at 1st BASE DNA Sequencing Division, Malaysia. The obtained sequences were compared with related sequences in the GenBank database using BLAST program.

#### Phosphate Solubilization

Phosphate solubilizing activity of all isolates was determined on Pikovskaya (PVK) agar (Pikovskaya, 1948) containing 0.5% (w/v) tricalcium phosphate. The agar plug (5 mm diameter) of actinobacterial growth on ISP medium 2 agar was transferred to PVK agar plates and incubated at 28 ± 0.2°C for 7 days. The occurrence of a clear zone around the agar plug was considered a positive indication of phosphate solubilization (Nautiyal, 1999). Quantitative analysis of tricalcium phosphate solubilization in liquid medium was also carried out using the Fiske and Subbarow (1925). Briefly, 5 agar plugs (5 mm diameter) of fully grown actinobacteria were inoculated into 50 ml of Pikovskaya’s broth and were incubated with shaking at 120 rpm for 7 days at 28 ± 0.2°C. The supernantant was collected by centrifugation at 12,000 rpm for 15 min. The resultant supernatant (500 μl) was mixed with 500 μl of 10% (w/v) trichloroacetic acid in a test tube. Four milliliters of the color reagent (1:1:1:2 ratio of 3M H_2_SO_4_, 2.5% ammonium molybdate (w/v), 10% ascorbic acid (w/v) and sterile distilled water) was added and the mixture was incubated at room temperature for 15 min. The pH value of the culture broth was also measured on day 0 and day 7. Sterile PVK medium served as a control. The optical density was read at 820 nm using a spectrophotometer (GENESYS TM 20 Visible Spectrophometer, Thermo Fishcer Scientific). Soluble phosphorus was estimated using a standard curve.

#### Siderophore Production

Siderophore production in all isolates was determined by chrome azurol S (CAS) assay (Schwyn and Neilands, 1987). Actinobacteria were grown on ISP medium 2 agar for 5 days at 28 ± 0.2°C. Agar plugs (5 mm diameter) of actinobacterial growth that were grown on ISP medium 2 agar were transferred to CAS agar and incubated for 7 days at 28 ± 0.2°C in a dark room. A yellow to orange zone around the actinobacterial disks indicated siderophore production. Siderophore quantity and types were determined and quantified by ferric perchlorate assay (Atkin et al., 1970) for hydroxamate type and Arnow assay (Arnow, 1937) for catecholate type.

### *In Vitro* Assay for Drought Tolerance in Actinobacteria

All actinobacteria were grown on ISP medium 22 agar for 7 days. Agar plugs (5 mm diameter) of fully grown actinobacteria were transferred to 10% Tryptic soy agar (TSA) that was supplemented with 0 gL^-1^; 85 gL^-1^; 285 gL^-1^; 405 gL^-1^; 520 gL^-1^, and 660 gL^-1^ of sorbitol to adjust the a_w_ value of the media in order to simulate water stress at 40°C for 7 days. The final a_w_ value of the media were 0.998, 0.986, 0.957, 0.919, 0.897, and 0.844, respectively (Hallsworth et al., 1998). Growth recorded at a_w_ of 0.919 was considered a drought-tolerant property.

### Growth Promotion of *Vigna radiata* by Selected Actinobacteria

Actinobacteria isolate S3 was selected based on its *in vitro* plant growth promoting properties. Mung beans (*Vigna radiata*) were used as a representative of dicotyledon plants. Mung bean seeds (Raithip Brand, Thai Cereal World Company, Thailand) were surface sterilized by being immersed sequentially in 2% (v/v) sodium hypochlorite for 1 min, 95% (v/v) ethanol for 1 min, 70% (v/v) ethanol for 1 min and then washed with sterile distilled water for 1 min (five times). Five decontaminated seeds were randomly selected to check for surface sterility on nutrient agar. Cell suspension was prepared from 7-day-old culture of isolate S3 grown in ISP medium 2 broth. Surface sterile seeds were mixed with 10^8^ CFU ml^-1^ cell suspension in 25 ml sterile water on a shaker at 120 rpm for 3 h before being sown in a sand pot. The following treatments, each with three hundred replicates of mung bean seeds, were conducted: (1) control (without bacterial inoculation), (2) cell suspension of isolate S3 (10^8^ CFU ml^-1^), and (3) standard IAA solution adjusting the concentration to 11.12 μg ml^-1^. The experiment was carried out in 2.5 cm × 2.5 cm pots and kept in a greenhouse at 30°C for 7 days. Pots were arranged in a completely randomized arrangement. After 7 days, the following growth parameters were recorded: fresh weight (g), root length (cm), seed germination (%) and total length (cm). Seed germination was calculated as a percentage of the number of germinated seeds from a total of 100 sown seeds per pot.

### Growth Promotion of Rice Under Drought Conditions in Low Nutritional Soil by Selected Actinobacteria

In this experiment, Thai jasmine rice (*Oryza sativa*) KDML105 was used as a representative monocotyledon plant. The soil used in the experiment was of a sandy loam type and had poor nutritional value. The soil was bought from Lan Sai Company, Chiang Mai, Thailand. Soil properties were determined by Central Laboratory, Faculty of Agriculture, Chiang Mai University. The soil was sterilized by being autoclaved twice for 20 min over two consecutive days. The sterility of the soil was determined by spreading the autoclaved soil suspension on ISP medium 2 and nutrient agar.

Rice seeds were kindly supplied by Chiang Mai Rice Seed Center, Rice Seed Division, Rice Department, Ministry of Agriculture and Cooperatives, Thailand. Seeds were surface sterilized by being immersed sequentially in 2% (v/v) sodium hypochlorite for 1 min, 95% (v/v) ethanol for 1 min and 70% (v/v) ethanol for 1 min, and then being washed with sterile distilled water for 1 min (three times). Five decontaminated seeds were randomly selected to check for surface sterility on ISP medium 2 and nutrient agar. Surface sterile seeds were mixed with 10^8^ CFU ml^-1^ of isolate S3 and incubated on a rotary shaker at 120 rpm at 30°C for 16–18 h before being sown. Seedlings were prepared by sowing 100 surface sterile seeds in a tray (25 cm × 35 cm × 9 cm) containing 1 kg of sterile soil. Seedlings were watered once a day with sterile distilled water for 7 days at room temperature. Seed germination was calculated as a percentage of the number of germinated seeds from a total of 100 sown seeds per tray. Seven-day-old seedlings having two leaves were transferred to a bigger pot for further growth promoting experimentation.

The experiment was carried out in pots (20 cm diameter × 15 cm height) containing 3 kg of sterile soil and kept in a greenhouse at ambient light and temperature for 45 days with five replicates per treatment and five seedlings per pot (*n* = 25). Pots were arranged in a completely randomized arrangement in a greenhouse. Tap water was supplied once a day to full container capacity and kept at 1 cm above soil level. Fertilizer was not added in order to maintain the low nutritional value of the soil. The following treatments were investigated: (1) control (without bacterial inoculation) and (2) drought-induced treatment [seeds mixed with cell suspension of isolate S3 (10^8^ CFU ml^-1^)]. In addition, a well-watered treatment [seeds mixed with cell suspension of isolate S3 (10^8^ CFU ml^-1^)] with its control (without bacterial inoculation) was also carried out for comparison purposes. Drought stress was induced by completely withholding water starting on day 36 for 10 days. At the end of the experiment (day 46 after being sown), the following growth parameters were recorded: fresh weight (g), dry weight (g), root length (cm), total length (cm), height (cm), stem diameter (mm), number of leaves, relative water content (RWC), chlorophyll content (mg g^-1^) and proline content (μmol g^-1^). Proline content was determined by rapid colorimetric method (Bates et al., 1973). Chlorophyll content was determined spectrophotometrically based on the standard method of Arnon (1949). RWC of rice leaves (*n* = 5) was calculated from the formula RWC (%) = [(FM-DM)/(TM-DM)] × 100 according to Oukarroum et al. (2007).

Colonization of rice root by isolate S3 was confirmed by dilution spread-plate and microscope examination. Briefly, the root was ground in TE buffer and shaken on a rotary shaker for 3 h at 180 rpm. The root suspension (1 ml) was serially diluted and spread on ISP medium 2 agar that was supplemented with 25 μl/ml nalidixic acid and 100 μg/ml ketoconazole. The suspension was then incubated at 30°C for 7 days. 16S rRNA gene sequencing was also performed on the obtained isolate.

Isolate S3 was also screened for 1-aminocyclopropane-1-carboxylate (ACC) deaminase production as described by Palaniyandi et al. (2014). Inoculation was done on DF minimal salts agar (Dworkin and Foster, 1958) with no nitrogen source and DF minimal salts agar that had been supplemented with either 5 mmol l^-1^ of ammonium sulfate or ACC. Plates were then incubated at 30°C for 10 days. The level of growth of the isolates on three different media was compared. Strains that were able to utilize ACC as a nitrogen source (ACC deaminase positive) exhibited a level of growth equal to the growth on (NH_4_)_2_SO_4_ containing medium, whereas strains that could not utilize ACC showed no growth, which was compatible to that of the medium without a nitrogen source.

### Nucleotide Sequence Accession Numbers

The 16S rRNA gene sequences of all actinobacterial isolates were deposited in the DDBJ, EMBL and GenBank nucleotide sequence databases under accession numbers LC062606-LC062608, LC065388-LC065389 and LC207997.

### Statistical Analysis

The data were analyzed using one-way analysis of variance (ANOVA) and Tukey’s multiple range tests (TMRT). The detection for all treatments in this study was calculated using SPSS (version 16.0) at *p* = 0.05.

## Results

### Selective Isolation of Actinobacteria From *Funneliformis mosseae* Spores

In this section, we tried to isolate cultivable actinobacteria that are associated with the spores of arbuscular mycorrhiza, *Funneliformis mosseae*. The first attempt at isolation without employing an enrichment step in the soil extract broth resulted in no actinobacterial growth on both selective media. Six presumptive actinobacteria were successfully isolated from the spores of *F. mosseae* using an enrichment step. Isolates 48, S1 and S3 were recovered from starch casein agar, whereas the remaining isolates were grown on humic acid vitamin agar (**Table 1**). The total actinobacterial count was 5 × 10^2^ CFU/20 spores on the starch casein agar. No microorganisms were grown on the nutrient agar and the ISP medium 2 agar obtained from the final washing water.

**Table 1:** Identification of actinobacteria associated with *F. mosseae*’s spores based on 16S rRNA gene sequence analysis.

Isolates no.	Isolation media	Length (bp)	Accession number	Similarity (%)	Closest match
48	SC	1412	LC062606	99.86	*Streptomyces thermocarboxydus* DSM 44293^T^
S1	SC	1392	LC207997	99.28	*Streptomyces spinoverrucosus* NBRC 14228^T^
S3	SC	1410	LC062607	99.93	*Streptomyces thermocarboxydus* DSM 44293^T^
S4	HV	1200	LC062608	99.75	*Streptomyces pilosus* NBRC 127772^T^
S4-1	HV	1422	LC065389	99.79	*Streptomyces pilosus* NBRC 127772^T^
SP	HV	1421	LC065388	98.56	*Pseudonocardia nantongensis* KLBMP 1282^T^

*SC, starch casein agar; HV, humic acid-vitamin agar*.

### Identification of Actinobacteria Based on 16S rRNA Gene Analysis

**FIGURE 1:**
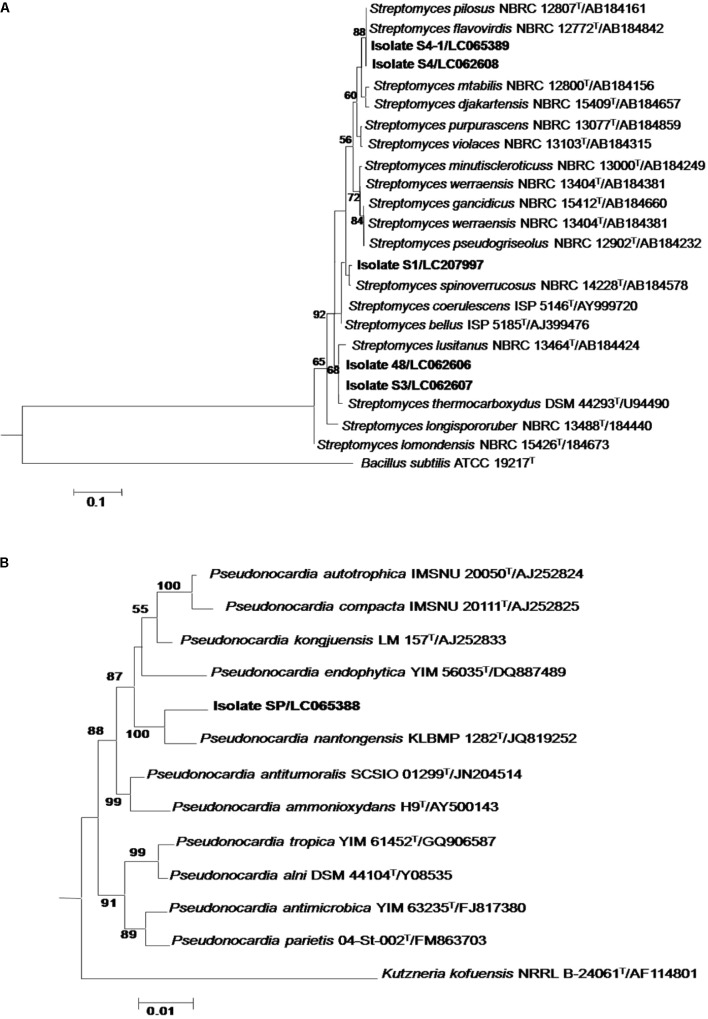
Neighbor-joining phylogenetic tree based on an almost complete 16S rRNA gene sequence of *Streptomyces* **(A)** and *Pseudonocardia* **(B)** isolates representative of their related taxa. Bootstrap values are based on 1,000 re-samplings. Only values of >50% are shown at the nodes.

The 16S rRNA genes obtained from all isolates were amplified with primers 27F and 1525R and sequenced. The resultant sequences ranged from 1200 to 1422 bp were identified using the BLAST program in the EzBioCloud database. Their closest phylogenetic neighbors are shown in **Table 1**. Five isolates were identified as members of the genus *Streptomyces*. Isolates 48 (99.86% similarity) and S3 (99.93% similarity) were closely related to *S. thermocarboxydus* DSM 44293^T^, whereas isolates S4 and S4-1 shared 99.75% and 99.79% similarity with *S. pilosus* NBRC 127772^T^. Isolate S1 was closely related to *S. spinoverrucosus* NBRC14228^T^ (99.28% similarity). The remaining isolate, isolate SP was identified as *Pseudonocardia* and closely related to *P. nantongensis* KLBMP 1282^T^ (98.56% sequence similarity). Phylogenetic analysis based on an almost complete sequence of the *Streptomyces* isolates is showed in **Figure 1A**. Isolates 48 and S3 were in the *S. thermocarboxydus* clade and supported by 88% bootstrap value, whereas isolates S4 and S4-1 fell into the *S. pilosus* clade with 68% bootstrap support. Isolate S1 was clustered with *S. spinoverrucosus*. The *Pseudonocardia* isolate SP shared a clade with *P. nantongensis* KLBMP 1282^T^, a relationship supported by a 100% bootstrap value (**Figure 1B**). The sequences from all isolates were deposited in the DDBJ database under the accession numbers shown in **Table 1**.

### *In Vitro* Plant Growth Promoting Potential

#### IAA Production

All 5 isolates of *Streptomyces* (48, S1, S3, S4, S4-1) were positive for IAA production in a range of 0.74 – 11.12 μg ml^-1^ (**Table 2**). Only the *Pseudonocardia* isolate SP showed a negative result. Isolate S3 produced the highest IAA value of 11.12 μg ml^-1^. This isolate also produced 1.12 μg ml^-1^ without L-tryptophan supplement. In addition, during periods of reduced water activity, isolate S3 still produced IAA at up to a_w_ = 0.919 (data not shown). TLC analysis of the IAA sample showed a pink color spot at similar Rf when compared with the chemical IAA standard (Supplementary Figure S1). PCR amplification of the IAA gene in isolate S3, the highest IAA producer, showed a product of approximately 1600 bp and was similar to that of the positive control, *S. coelicolor* A3(2). Sequence analysis of this PCR fragment using the BLAST program (blastx) revealed a similarity to the amino oxidase gene in *Streptomyces coelicolor* A3(2) (NP_625735).

**Table 2:** Indole-3-acetic acid (IAA) production, phosphate solubilization and siderophore production of actinobacteria associated with *F. mosseae*’s spores.

Isolate	IAA production (μg ml^-1^)	Phosphate solubilization			Siderophore production		
		Clear zone on PVK agar (mm)	P released in PVK broth (mg L^-1^)	pH	Color zone on CAS agar (mm)	Hydroxamate-Type (μmol L^-1^)	Catecholate-Type (μmol L^-1^)
48	4.44 ± 0.04	5.17 ± 0.29	217.56 ± 0.56	5.09 ± 0.09	17.83 ± 0.58	7.37 ± 0.009	17.50 ± 0.008
S1	9.08 ± 1.79	5.60 ± 0.76	215.60 ± 0.65	5.53 ± 0.72	13.00 ± 0.00	97.5 ± 3.63	7.54 ± 3.50
S3	11.12 ± 0.02	7.00 ± 0.50	224.27 ± 0.76	4.46 ± 0.25	15.83 ± 0.76	5.09 ± 0.003	39.17 ± 0.002
S4	0.74 ± 0.00	0	0	0	13.50 ± 1.50	1.40 ± 0.001	30.87 ± 0.005
S4-1	6.04 ± 0.05	0	0	0	14.00 ± 0.50	1.05 ± 0.000	17.50 ± 0.001
SP	0	0	0	0	13.83 ± 0.29	12.45 ± 0.003	2.12 ± 0.006

*PVK, Pikovskaya’s agar; CAS, Chrome Azurol Sulphonate agar. Data represent mean values of three replicates ± SD. Different letters indicate the existence of significant differences according to Tukey test (*P* = 0.05)*.

#### Phosphate Solubilization

Half of the isolates (isolates 48, S1, S3) were found to produce clear zones around their colonies on Pikovskaya’s agar (**Table 2**). The largest clear zone diameter of 7.00 ± 0.05 mm was observed (Supplementary Figure S2a). Quantitative estimation of phosphate solubilization in the culture broth was in a range of 215.6 – 224.27 mg L^-1^, with the highest value obtained from isolate S3 (224.27 ± 0.06 mg L^-1^). A decline in the pH value of the culture filtrate was observed in all these positive isolates. The lowest pH value was recorded at pH 4.46 ± 0.25 for isolate S3.

#### Siderophore Production

All isolates could produce siderophore on CAS agar as an orange halo zone was observed around the agar plugs (**Table 3**). Isolate 48 produced the largest zone of siderophore production (17.83 ± 0.58 mm) followed by isolate S3 (15.83 ± 0.58 mm) (Supplementary Figure S2b). All isolates could produce both catecholate and hydroxamate type siderophores at various levels. Isolate S3 produced the highest catecholate type siderophore (39.17 ± 0.002 μmol L^-1^), whereas isolate S1 produced the highest hydroxamate type siderophore (97.50 ± 3.63 μmol L^-1^) (**Table 2**).

**Table 3:** Growth of actinobacteria associated with *F. mosseae*’s spores under reduced water activity.

Sorbitol (g/L)	Water activity (a_w_)	Isolates					
		48	S1	S3	S4	S4-1	SP
0	0.998	+	+	+	+	+	+
85	0.986	+	+	+	+	+	+
285	0.957	+	+	+	+	+	+
405	0.919	-	+	+	-	-	-
520	0.897	-	-	-	-	-	-
660	0.844	-	-	-	-	-	-

*+, growth; -, no growth*.

### *In Vitro* Assay for Drought Tolerance in Actinobacteria

Two *Streptomyces* isolates, S1 and S3, showed a high tolerance to drought *in vitro*, as they were able to grow in medium with reduced water availability (a_w_ = 0.919). The remaining isolates could grow up to a_w_ of 0.957 (**Table 3**). Though both isolates could grow under water stressed conditions, isolate S3 was selected as a candidate for rice growth promotion under drought conditions due to its better performance with regard to *in vitro* plant growth promoting properties.

### Growth Promotion of *Vigna radiata* by Selected Actinobacteria

**FIGURE 2:**
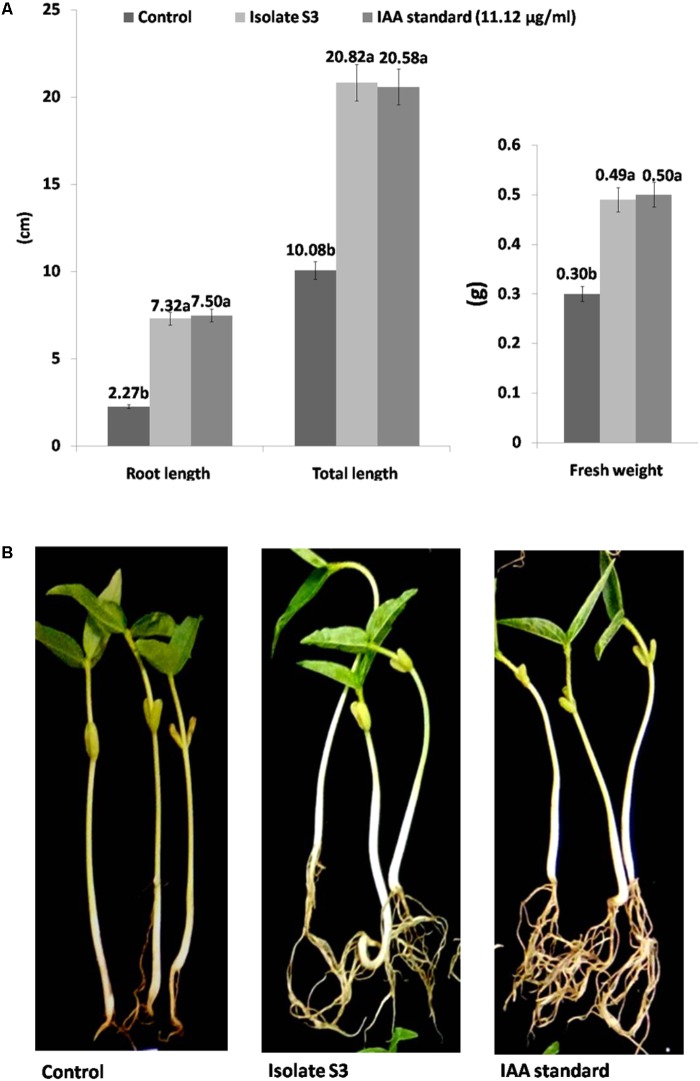
Growth promotion of mung beans (*Vigna radiata*) by *S. thermocarboxydus* isolate S3 **(A)**, root intensity **(B)**.

The effects of *S. thermocarboxydus* isolate S3 inoculation on mung bean seeds were observed. The percentage of seed germination in all treatments involving isolate S3 was in a range of 95–98%, which was statistically higher than the control. The highest value of enhancement of all growth parameters was obtained from Treatments 2 and 3 (**Figure 2A**). Treatments with isolate S3 showed higher fresh weight, root length and total length when compared to the control. The increases in fresh weight, root length and total length were not significantly different in Treatments 2 and 3. In addition, though root intensity was not directly observed, it is evident from **Figure 2B** that Treatments 2 and 3 displayed more intense roots than the control.

### Growth Promotion of Rice Under Drought Conditions in Soil With Low Nutritional Value by Selected Actinobacteria

**FIGURE 3:**
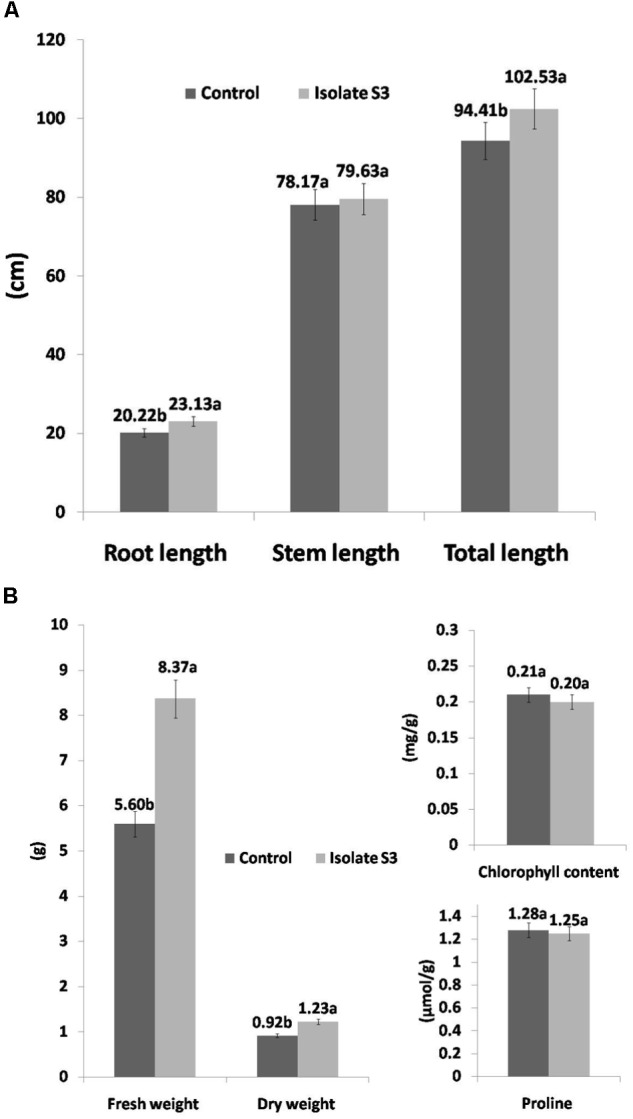
Growth promotion of Thai jasmine rice (*O. sativa*) KDML105 by *S. thermocarboxydus* isolate S3 under well-watered conditions. **(A)** Root, stem and total length. **(B)** Fresh weight, dry weight, chlorophyll and proline contents.

**FIGURE 4:**
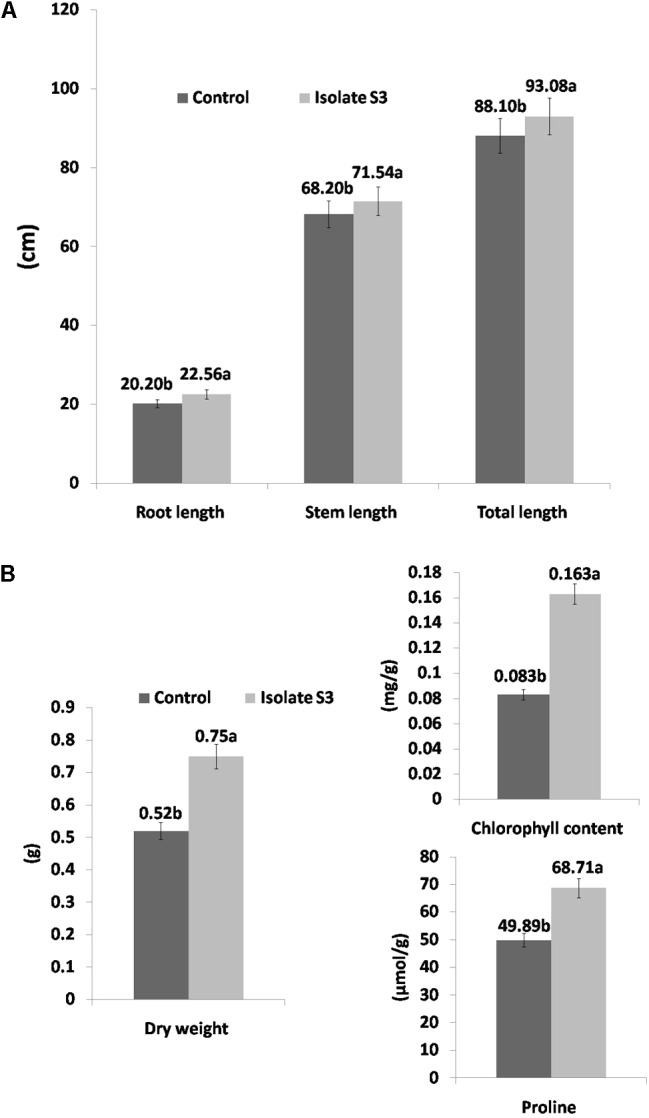
Growth promotion of Thai jasmine rice (*O. sativa*) KDML105 by *S. thermocarboxydus* isolate S3 under drought conditions. **(A)** Root, stem, and total length. **(B)** Dry weight, chlorophyll and proline contents.

**FIGURE 5:**
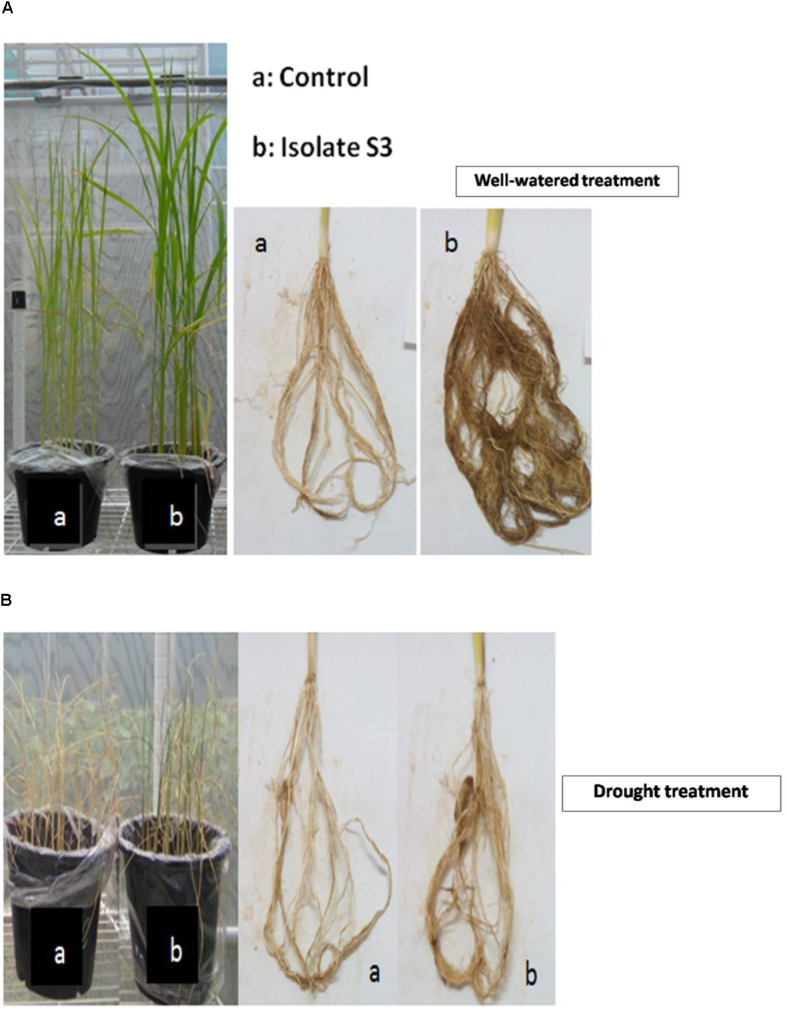
Growth of Thai jasmine rice (*O. sativa*) KDML105 inoculated with *S. thermocarboxydus* isolate S3 under well-watered conditions **(A)** and drought **(B)**. a: control; b: isolate S3.

The soil used in this study was of poor quality as can be seen in **Table 4**. The organic matter content was only 0.75%, which determined it to be a low nutritional type of soil for rice cultivation as recommended by the Rice Department, Ministry of Agriculture and Cooperatives, Thailand. In addition, the nitrogen, phosphate and potassium levels were also low and this was especially true for nitrogen. The effects of *S. thermocarboxydus* isolate S3 inoculation on Thai jasmine rice KDML105 is presented in **Figures 3**–**5**. Seed germination was recorded between 98 and 99% for all treatments, which was higher than the control (96%). In the well-watered treatment, the positive effects of *S. thermocarboxydus* isolate S3 was seen in the rice as indicated by the following observed growth parameters, namely fresh weight, dry weight, root length and total length, which were all significantly higher than in the control (**Figure 3A**). However, the chlorophyll and proline contents were similar between the two treatments (**Figure 3B**). The RWC value of the rice leaves obtained from the treatment using isolate S3 was 96.1% compared to 81.0% in the control.

**Table 4:** Characteristics of soil used in plant growth promotion experiment.

No	Properties	Amount
1	Electrical Conduction (μS/cm)	56.80
2	Exchangeable Potassium (mg/kg)	79.83
3	Organic Matter (%)	0.75
4	Total Nitrogen (%)	0.04
5	Available Phosphate (mg/kg)	10.20
6	Exchangeable Magnesium (mg/kg)	98.12
7	Available Iron (mg/kg)	35.04
8	Extractable Sulfur (mg/kg)	23.93

For the drought-induced treatment, a similar level of growth enhancement was observed in all tested parameters. The root, stem and total length measurements of the rice in *S. thermocarboxydus* isolate S3 treatment were higher than in the control (**Figure 4A**). The proline and chlorophyll contents, as well as the fresh and dry weight, were also higher than in the control (**Figure 4B**). At the end of the experiment, all rice plants in the control treatment withered and died, whereas in the treatment with *S. thermocarboxydus* isolate S3, some plants were still green (**Figures 5A,B**). The RWC value of the rice leaves in the inoculated rice with isolate S3 was 48.3% as compared to 43.3% in the control under drought stress conditions. *S. thermocarboxydus* isolate S3 was re-isolated from the rice root after harvesting. A count of 2 × 10^3^ cfu/g fresh weight was obtained. The re-isolated strain showed 100% 16S rRNA gene sequence, which was identical to that of the *S. thermocarboxydus* isolate S3. In addition, filamentous cells of *S. thermocarboxydus* isolate S3 were observed under a light microscope as is shown in the Supplementary Figure S3. ACC deaminase activity was detected in isolate S3 as it could grow on DF salt minimal agar that was supplemented with 5 mmol l^-1^ of ACC as the nitrogen source.

## Discussion

### Isolation and Identification of Actinobacteria From Spores of *Funneliformis mosseae*

Cultivable actinobacteria associated with arbuscular mycorrhizal spores were reported to be an interesting source of both biocontrol and plant growth-promoting activities (Schrey et al., 2012; Mohandas et al., 2013; Poovarasan et al., 2013; Battini et al., 2016). These findings encouraged us to explore actinobacteria that was obtained from the spores of *Funneliformis mosseae*. In the present study, we successfully isolated 6 actinobacteria from the spores of *Funneliformis mosseae*. The predominant isolates were members of the genus *Streptomyces*, a finding consistent with previous reports (Schrey et al., 2012; Mohandas et al., 2013; Poovarasan et al., 2013; Battini et al., 2016). The fact that we could isolate actinobacteria from surface sterilized spores suggested that they should be obtained from inside the spores. No bacterial growth was observed on nutrient agar and ISP2 agar obtained from the final wash water. These findings suggested that these actinobacteria are likely to be endophyte.

The interaction between actinobacteria and their mycorrhizal symbiosis have not been well-documented. Most research studies were conducted on Gram-negative bacteria such as *Pseudomonas*. However, several researchers have reported on the enhanced spore germination or mycorrhiza formation of various AM fungi in the presence of actinobacteria (Frey-Klett et al., 2007). Tylka et al. (1991) found that volatiles obtained from S. *orientalis* stimulated the germination of both *Gigaspora margarita* and *G. mosseae* spores on water agar. Similarly, the spore germination of *G. margarita* was found to be stimulated by volatiles of soil-isolated actinobacteria, especially those with straight spore chains and 2-methylisoborneol (MIB) producers (Carpenter-Boggs et al., 1995). The same authors speculated that the stimulation of AM spore germination enhanced the probability of the formation of mycorrhizal associated with nearby plants. Inoculation of *S. coelicolor* 2389 with *G. intraradices* increased the intensity of mycorrhizal root colonization and arbuscular formation in sorghum (Abdel-Fattah and Mohamedin, 2000). *Streptomyces* was also found to enhance mycorrhiza formation in the ectomycorrhizal fungus of *Amanita muscaria* as a result of the promotion of fungal growth (Schrey et al., 2005). In addition, mycorrhiza-associated bacteria together with AM fungi contributed to protection against root pathogens (Frey-Klett et al., 2007). For example, mycorrhizal associated streptomycetes obtained from Norway spruce trees produced secondary metabolites displaying antifungal activity against the growth of several plant pathogenic fungi including *Fusarium oxysporum* (Schrey et al., 2012). *Streptomyces* obtained from *G. mossaea* spores also showed strong antifungal activity against *F. oxysporum* and *Alternaria solani* (Mohandas et al., 2013). Considering the plant growth promoting ability of the isolated actinobacteria, it is possible that these actinobacteria are “mycorrhiza helper bacteria” that can contribute to nutrient mobilization from the soil and impact root structure through the production of growth promoters, as has been suggested by Frey-Klett et al. (2007). However, the possibility of the isolated actinobacteria being involved in the process of promoting mycorrhization could not be ruled out and requires further study.

It is interesting to note that a member of the genus *Pseudonocardia* was isolated from mycorrhiza spores for the first time in this study. It was previously only detected in the spores of *Gigaspora margarita* and *Gigaspora rosea* by DGGE (Long et al., 2008). Currently, DGGE analysis of *F. mosseae* spores has revealed the presence of actinobacteria in the genera *Amycolatopsis*, *Arthrobacter*, and *Propionibacterium* (Agnolucci et al., 2015). *Glomus mosseae* spores were reported to harbor cultivable actinobacteria of the genus *Arthrobacter, Curtobacterium, Liefsonia, Microbacterium, Micrococcus*, and *Streptomyces* (Bharadwaj et al., 2008b; Mohandas et al., 2013; Poovarasan et al., 2013; Selvakumar et al., 2016).

It is also worth mentioning that we could not isolate any actinobacteria without an enrichment step in the soil extract solution. Enrichment is a common practice for effectively increasing the population of target microorganisms (Kamagata, 2015). Soil extract was successfully used to isolate novel actinomycetes from the soil (Hamaki et al., 2005). Conventional cultures and isolation media that are generally nutritionally rich usually result in a failure to cultivate most bacteria in nature. Our results suggest that the addition of the soil extract could support the growth of actinobacteria, which represents a small proportion of the bacteria that is associated with mycorrhizal spores and can increase the numbers to a detectable level on the isolation plate.

It is evident from the 16S rRNA gene sequences and the phylogenetic analysis that 5 isolates were members of the genus *Streptomyces*. These isolates were closely related to *S. thermocarboxydus*, *S. pilosus*, and *S. spinoverrucosus* (**Table 1** and **Figure 1A**). Based on this data, we identified isolates 48 and S3 as *S. thermocarboxydus*, isolates S4 and S4-1 as *S. pilosus* and isolate S1 as *S. spinoverrucosus*. These three *Streptomyces* species have never been isolated from mycorrhizal spores. Isolate SP was identified as *Pseudonocardia* and is closely related to *P. nantongensis* KLBMP 1282^T^ (**Table 1**). It shared a well support branch with *P. nantongensis* KLBMP 1282^T^ (**Figure 1B**). To the best of our knowledge, this is the first *Pseudonocardia* strain isolated from mycorrhizal spores. This strain shared only 98.56% similarity with *P. nantongensis* KLBMP 1282^T^, a value below the proposed 98.7% (Stackebrandt and Ebers, 2006) or 98.65% (Kim et al., 2014) cut-off values for the delineation of novel species. It is likely that isolate SP will represent a novel species within the genus *Pseudonocardia* though a further characterization using the polyphasic approach is needed. However, the detailed formal description of this strain as a novel species will be the subject of another publication.

### *In Vitro* Plant Growth Promoting Activities

Indole-3-acetic acid (IAA) is a phytohormone of the auxin type, which helps promote plant growth and development especially in the root system. The production of IAA is common in actinobacteria. Several *Streptomyces* species are known to produce IAA including *S. coelicolor*, *S. griseus*, or *S. scabies* (Manulis et al., 1994). A high proportion (70%) of IAA producing actinobacteria that are associated with the mycorrhizal spores of *R. intraradices* was also reported (Battini et al., 2016). In the present study, all *Streptomyces* isolates were able to produce IAA in the presence of L-tryptophan (0.74 – 11.12 μg ml^-1^, **Table 2**). *Streptomyces* and *Leifsonia poae* isolated from *G. mosseae* spores were reported to produce IAA at similar levels to our isolates (5.0–10.1 μg ml^-1^) (Mohandas et al., 2013). A higher level of IAA production of 43.8 μg ml^-1^ compared to *S. thermocarboxydus* isolate S3 (11.12 μg ml^-1^) was recorded in *S. thermocarboxydus* DBT219 using endophytic actinobacteria associated with tomatos (*Solanum lycopersicum*) (Passari et al., 2016). However, those authors did not indicate the amount of supplemented L-tryptophan in the experiment. *Streptomyces* isolates obtained from Thai medicinal plants produced IAA in a range of 11-144 μg ml^-1^, while *S. viridis* CMUH-009 could produce IAA at a level as high as 300 μg ml^-1^ under optimized conditions (Khamna et al., 2010). The expected PCR product of approximately 1,600 bp from *S. thermocarboxydus* isolate S3, as well as *S. coelicolor* A3(2), indicated the presence of the IAA encoding gene. However, BLAST identification of this PCR product as an amino oxidase gene in isolate S3 suggested that this *Streptomyces* may use the tryptamine (TAM) pathway in IAA synthesis instead of the indole-3-acetamide (IAM) pathway, as was the case with several *Streptomyces* species including *S. coelicolor* A3(2) and *S. scabies* (Manulis et al., 1994).

Several actinobacteria associated with mycorrhizal spores were reported to have phosphate solubilization properties. Three of our *Streptomyces* isolates, namely isolates 48, S3 and S1, produced a clear zone of tricalcium phosphate solubilization on Pikovskaya’s agar. *Streptomyces* and *Leifsonia* isolated from *G. mosseae* spores were also found to produce clear zones of tricalcium phosphate solubilizing activity (Mohandas et al., 2013). Similarly, *Arthrobacter ilicis* obtained from the spores of *G. intraradices* could solubilize phosphate on Pikovskaya’s agar (Bharadwaj et al., 2008a). However, the quantitative determination of phosphate solubilization in the culture broth was not reported in these previous research studies. High phosphate solubilization in the range of 215.6 – 224.27 mg L^-1^ as was recorded in this study was higher than that of the *Streptomyces* isolates obtained from the sediment of Chorao Island, India (89.3 – 112.1 μg mL^-1^) (Dastager and Damare, 2013). Nevertheless, a higher degree of phosphate solubilizing activity of 72.13 mg/100 ml was reported in *Streptomyces* obtained from the wheat rhizosphere soil in Pakistan (Anwar et al., 2016). The ability to solubilize phosphate in actinobacteria obviously varies from strain to strain and species to species, as exemplified by our results and the previous research reports.

The acidification of the culture filtrate was observed in all isolates that could release soluble phosphate in the culture broth. This observation suggested that the solubilization process was involved with the production of organic acids. Microbial solubilization of mineral phosphates is proposed to either be due to the production of organic acids (Surapat et al., 2013) or the production of chelating substances such as siderophores (Hamdali et al., 2008). A preliminary investigation on the quality and quantity of organic acids in the culture broth by HPLC analysis confirmed the presence of several organic acids including gluconic acid, malonic acid, oxalic acid and propionic acid (data not shown). Lactic and 2-ketogluconic acids were the predominant acids found in phosphate-solubilizing *Streptomyces* (Banik and Dey, 1982). Additionally, phosphate-solubilizing actinobacteria, *Micromonospora endolithica*, was reported to be capable of producing organic acids namely citric, oxalic, gluconic, malic, succinic, acetic, and lactic acids (El-Tarabily et al., 2008).

All actinobacteria that were isolated from the spores of *F. mosseae* were able to produce siderophores in both hydroxamate and catecholate types at varying concentrations. Siderophores are small molecules of iron chelating compounds produced by several microorganisms including actinobacteria under iron starvation (Wang et al., 2014). Siderophores are known to promote plant growth and inhibit plant pathogens (Verma et al., 2011; Sadeghi et al., 2012). Several *Streptomyces* species have been found to be able to produce desferrioxamine (hydroxamte type siderophores) including *S. pilosus* (Wang et al., 2014). Catecholate types are not common among siderophore-producing actinobacteria. However, *Streptomyces* were found to be able to secrete enterobactin, a catecholate type siderophore of *Enterobacteriaceae* (Lee et al., 2012). All actinobacteria obtained from *G. mosseae* spores including *Streptomyces* were also able to produce siderophores on CAS agar (Mohandas et al., 2013). Similarly, 79% of the cultivable actinobacteria obtained from *R. intraradices* spores could produce siderophores (Battini et al., 2016). The siderophore-producing endophytic *Streptomyces* was clearly shown to be able to promote the growth of Thai jasmine rice by significantly increasing root and shoot biomass, as well as root length, in comparison with the siderophore-deficient mutant (Rungin et al., 2012).

It is worth mentioning that three of our *Streptomyces* isolates (48, S1, S3) displayed all of the plant growth-promoting activities we investigated. The observation that the actinobacteria associated with the spores of *F. mosseae* are equipped with plant growth promoting properties *in vitro* suggests that these actinobacteria are potential candidates for *in planta* testing. This strategy has been used successfully to identify potential actinobacteria that can promote the growth of several plant species, e.g., guava (Mohandas et al., 2013), rice (Rungin et al., 2012; Gopalakrishnan et al., 2014) tomato (Passari et al., 2016) and wheat (Anwar et al., 2016).

### *In Vitro* Assay for Drought Tolerance in Actinobacteria

Bacteria in general prefer a high a_w_ value for growth. A minimum a_w_ for bacterial growth was defined at 0.900a_w_ for a majority of bacteria (Grant, 2004). In the present study, *Streptomyces* isolates S1 and S3 grew up to 0.919 a_w_ which could indicate xerotolerance. Drought is a water deficit condition that does not allow for the growth of most plants. To use plant growth promoting bacteria to alleviate drought stress for plants, one basic requirement is the growth of plant growth-promoting bacteria under such condition. The ability to grow during periods of low water availability is an important criterion for selecting potential actinobacterial isolates when testing the growth promotion of the plant under the conditions of drought. This screening approach was successfully used to obtain rhizosphere bacteria from cacti for the growth promotion of *Zea mays* L. under drought conditions (Kavamura et al., 2013). The ability of actinobacteria to grow at low a_w_ is not surprising as several species of actinobacteria, including several *Streptomyces* species, have been isolated from even the driest places on earth like the Atacama desert (Okoro et al., 2009). This report and our results strongly support the conclusion that these xerotolerant actinobacteria could survive under extreme water deficit environments and we recommended them as promising materials for plant growth-promoting bacteria under conditions of drought stress.

### Plant Growth Promotion

#### Growth Promotion of *Vigna radiata* by Selected Actinobacteria

This experiment was designed to test the ability of the selected *S. thermocarboxydus* isolate S3 to promote plant growth *in vivo*. Mung beans (*V. radiata*) have been widely cultivated throughout Southeast Asia. This plant species has been used as a test plant due to its short life cycle. The ability of isolate S3 to promote the growth of mung beans was evident (**Figure 2**). Similar results from treatment 2 (cell suspension and supernatant) and treatment 3 (addition of 11.12 μg ml^-1^IAA) indicated that the observed level of growth enhancement in the root and total length might be a result of the contribution of IAA that was produced by *S. thermocarboxydus* isolate S3 as this isolate was found to produce 11.12 μg ml^-1^ IAA *in vitro* (**Table 2**). More developed root systems were also observed in the treatment with isolate S3. This observation suggested the effect of IAA production by isolate S3. IAA is known for its effect on the production of lateral roots and the promotion of root-length (Etesami et al., 2015). Similar incidences of the promotion of plant growth that occurred due to the production of plant phytohormones by *G. mosseae* spores associated with actinobacteria in pomegranate seedlings have been reported (Poovarasan et al., 2013). *S. canus* which produced 10.10 μg ml^-1^ of IAA *in vitro* was found to increase shoot and root growth and plant dry matter in 6-month-old pomegranate seedlings. The production of bacterial phytohormones such as IAA is considered to be a major mechanism for drought endurance and resilience in microbial strains (Kaushal and Wani, 2016; Forni et al., 2017). This is the reason that *S. thermocarboxydus* isolate S3 was selected for further experimentation on rice growth promotion under induced drought conditions in the greenhouse.

#### Growth Promotion of Rice Under Drought Conditions in Soil With Low Nutritional Content by Selected Actinobacteria

It is evident that in soil having low nutritional content and under non-stress conditions, inoculation with isolate S3 improved the growth of rice seedlings as was observed in terms of root length, stem length, and fresh and dry weight (**Figure 3**). Our results provide evidence that the most prominent beneficial effects of inoculation with a potential PGPR is to be expected in poor soil when the development of the indigenous microbial community is inhibited (Ramos Solana et al., 2006). Similar results have been reported under other unfavorable conditions such as in rice (Yuwono et al., 2005) or wheat (Yandigeri et al., 2012) under conditions of drought and in tomatos (Palaniyandi et al., 2014) under conditions of salinity.

Rice is more susceptible to drought stress than most crop plants. According to the International Rice Research Institute (IRRI), when rice plants experience drought, they have a reduced ability to extract nutrients from the soil (Lafitte and Bell, 2017). Thai jasmine rice KDML105 is a dominant rice variety that has been widely cultivated in Thailand and is sensitive to drought (Cha-um et al., 2010). Therefore, it has been selected as a test plant in the investigation of the ability of plant growth promoting *S. thermocarboxydus* isolate S3 to protect KDML105 under conditions of induced drought stress in soil of low nutritional value. In this study, we induced conditions of drought stress in rice during the early vegetative stage, which requires large amounts of water for the development of a complete panicle formation^2^. This drought stress induces a reduction in rice growth and development as was observed in many growth parameters in this study. With the use of soil having low nutritional content in this study, the adverse effects on rice growth was expected to be more severe as was seen in reductions of fresh and dry weights (**Figures 3B**, **4B**).

The growth and development of rice was observed to decline due to the fact that drought stress induces damage to biochemical and physiological mechanisms (Pandey and Shukla, 2015). This stress could be alleviated through the application of a microbial inoculant that induces biochemical and physiological changes in plants to sustain their growth (Yang et al., 2009; Yandigeri et al., 2012; Vurukonda et al., 2016; Rubin et al., 2017). This study provides evidence that induced drought stress conditions could be relieved by inoculation with plant growth promoting *S. thermocarboxydus* isolate S3.

Fresh weight is another common morphological growth parameter that is severely affected by drought (Jaleel et al., 2009). Reduced biomass was reported in rice under conditions of water stress (Manickavelu et al., 2006; Farooq et al., 2009, 2010). Many studies have reported a significant decrease in the fresh and dry weights of the shoots (Centritto et al., 2009; Mostajeran and Rahimi-Eichi, 2009) and roots (Ji et al., 2012) under conditions of drought. Our results revealed a reduction in both the fresh (3.18 g) and dry (0.52 g) weights of drought-induced rice compared to well-watered rice in treatments without *S. thermocarboxydus* isolate S3 (5.6 g, 0.92 g). However, the fresh and dry weight measurements in drought-induced rice that has been inoculated with *S. thermocarboxydus* isolate S3 were higher than in the control. This observation implies the ability of plant growth promoting *S. thermocarboxydus* isolate S3 to reduce the damaging effects caused by drought stress.

Drought stress also affects various physiological processes in plants. One common adverse effect is on photosynthetic pigments. Chlorophyll is the most important pigment for photosynthesis in plants and thus affects their growth. Since severe drought stress may cause termination in photosynthesis. A reduction in chlorophyll content has been reported in KDML105 rice under conditions of drought stress (Cha-um et al., 2010) and in other rice varieties (Maisura et al., 2014; Yang et al., 2014). In the present study, the chlorophyll content in the well-watered treatment was similar between treatment with *S. thermocarboxydus* isolate S3 (1.25 mg/g) and the control (1.28 mg/g). However, a remarkable difference was observed in the drought-induced treatments. In the treatment with *S. thermocarboxydus* S3, the chlorophyll content (0.163 mg/g) revealed a 96.4% increase when compared to the control treatment (0.083 mg/g) without bacterial inoculation. This observation strongly suggests that *S. thermocarboxydus* isolate S3 could prevent the deleterious effects of drought on the chlorophyll content in Thai jasmine rice KDML105.

Proline accumulation in crop species has been established as a good indicator of water stress. Changes in proline concentrations in rice have been observed. Proline content in rice KDML105 increased under conditions of drought stress (Cha-um et al., 2010). An increase in proline levels is regarded as one mechanism of drought tolerance in certain plants including rice (Pandey and Shukla, 2015). Proline can act as both an osmo-protectant agent and a hydroxyl radical scavenger to protect plants from certain kinds of stress (Kaushal and Wani, 2016; Forni et al., 2017). It is evident from our results that *S. thermocarboxydus* isolate S3 could induce the accumulation of proline in Thai jasmine rice KDML105 that has been subjected to conditions of drought stress (**Figure 4B**).

Relative water content in plant leaves is considered one of the best criterion for measuring plant water status as drought is known to decrease the RWC in the leaves of plants under conditions of stress (Ngumbi and Kloepper, 2016). In this study, the leaves of rice seedlings inoculated with *S. thermocarboxydus* isolate S3 showed an 11% higher RWC than the un-inoculated leaves under drought conditions. Our results are in line with the previous findings by Grover et al. (2014) who observed that sorghum plants that were inoculated with PGPR *Bacillus* spp. strain KB129 showed a 24% increase in RWC over the control plants under drought stress conditions. Similar results were reported in maize seedlings that had been inoculated with several plant growth-promoting rhizobacteria (Sandhya et al., 2010; Vardharajula et al., 2011; Garcia et al., 2017). The data from those findings and this study suggest that plant growth promoting rhizobacteria could increase RWC and improve the survival rate of plants under conditions of drought stress. An increase in RWC should be considered an important drought tolerance strategy in plants.

Bacteria producing phytohormones such as auxins were reported to be able to improve the drought resistance of host plants (Yang et al., 2009; Vurukonda et al., 2016). Root growth enhancement by IAA promotes water and nutrient uptake, hence increase the tolerance of rice to conditions of drought (Dimpka et al., 2009). Root proliferation improvement in rice plants under conditions of drought stress has been shown to be a result of IAA production by inoculated rhizobacteria (Yuwono et al., 2005). *S. thermocarboxydus* isolate S3 was able to produce IAA at lower water availability (0.919a_w_; data not shown) which could promote the growth of rice seedlings under drought conditions. These results suggest that the level of drought tolerance observed in this study may be partly mediated by IAA.

Ethylene is a gaseous plant hormone, which is also known as a plant stress hormone. Under conditions of drought stress, plants produce ethylene to regulate homeostasis, which also causes reductions in root and shoot growth (Yang et al., 2009). 1-aminocyclopropane-1-carboxylate (ACC) is an immediate precursor of ethylene in higher plants. Bacteria containing enzyme ACC deaminase, which are able to degrade ACC to ammonia and α-ketobutyrate, could mitigate this deleterious effect of ethylene. ACC deaminase-producing bacteria confer tolerance against water deficiency in rice (Bal et al., 2013) and tomato plants (Palaniyandi et al., 2014).

The importance of IAA production and ACC deaminase activity in promoting plant-growth under conditions of drought has been recognized (Saleem et al., 2007; Dimpka et al., 2009; Kaushal and Wani, 2016; Ngumbi and Kloepper, 2016; Vurukonda et al., 2016). IAA is involved in ethylene biosynthesis through stimulation of enzyme ACC synthetase activity to convert *S*-adenosyl-methionine (SAM) to ACC (Saleem et al., 2007). Microbial uptake and hydrolysis of ACC by ACC deaminase-producing bacteria provide a level of equilibrium between the internal and external ACC levels. This lower ACC level reduced ethylene biosynthesis within the plant. In a relationship between IAA and the ethylene precursor, ACC supports the positive effects of IAA on root growth through a reduction of ethylene levels (Lugtenberg and Kamilova, 2009). El-Tarabily (2008) demonstrated that *Streptomyces filipinensis* no. 15, ACC-deaminase and an IAA producer, could reduce the endogenous levels of ACC, an ethylene precursor, in both the roots and shoots and subsequently enhanced tomato growth under greenhouse conditions. Similarly, ACC deaminase and IAA producing bacteria from the rhizosphere were shown to promote the growth of rice seedlings, especially with regard to root and shoot growth (Bal et al., 2013). Palaniyandi et al. (2014) reported on the plant growth-promoting and stress-alleviating activities from a halo-tolerant and ACC deaminase-producing *Streptomyces* sp. strain PGPA39 that was applied to tomato (*Solanum lycopersicum*) plants under conditions of salinity stress. Our results imply that *S. thermocarboxydus* isolate S3 uses its ability for IAA and ACC deaminase production as a mechanism to promote the growth of KDML105 rice under induced drought conditions.

The ability of *S. thermocarboxydus* isolate S3 to produce siderophores and solubilize phosphate is also beneficial for rice growth as phosphorus and iron are essential for plant growth. Enhanced plant nutritional uptake is an example of a direct mechanism that could stimulate plant growth (Bharadwaj et al., 2008a). *Streptomyces* from *G. mosseae* with phosphate solubilizing and siderophore-producing abilities were able to promote the growth of guava (Mohandas et al., 2013). Similarly, phosphate solubilizing rhizosphere *M. endolithica* was also able to promote the growth of *Phaseolus vulgaris* beans (El-Tarabily et al., 2008).

The successful re-isolation of *S. thermocarboxydus* isolate S3 from the roots along with the observation of the typical filamentous cells of *Streptomyces* on the root surface has suggested that this isolate has the ability to colonize the roots of rice. In addition, the substantial number of isolate S3 that was found in the roots after being harvested was suggestive of the survival of this strain within the root tissues. Similar results of bacterial colonization have been reported in previous studies (Etesami et al., 2014; Qin et al., 2017). This root colonization is an important factor in both plant-growth promoting activities and the survival of bacteria under conditions of abiotic stress such as drought (Gontia-Mishra et al., 2016).

## Conclusion

Our results provide evidence that actinobacteria were associated with arbuscular mycorrhizal spores of *F. mosseae*. One new genus of actinobacteria, *Pseudonocardia*, was added to the list of cultivable actinobacteria associated with arbuscular mycorrhizal spores as well as the species of *S. pilosus*, *S. spinoverrucosus*, and *S. thermocarboxydus*. The inoculation of *S. thermocarboxydus* isolate S3 could promote the growth of Thai jasmine rice in soil of low nutritional content and under induced conditions of drought stress. The positive effects of *S. thermocarboxydus* isolate S3 inoculation may be due to its PGP properties, in particular IAA production and ACC deaminase activity. The ability of this organism to produce plant growth promoting agents supports the possibility of using this actinobacteria for agricultural purposes especially in areas affected by water deficit stress or in arid and semi-arid habitats.

## Author Contributions

WP-A, KL, and SL designed the research and project outline. KL performed all the experiments. KL, ST, SL, and WP-A drafted the manuscript. All authors read and approved the final manuscript.

## Conflict of Interest Statement

The authors declare that the research was conducted in the absence of any commercial or financial relationships that could be construed as a potential conflict of interest. The handling Editor and reviewer SQ declared their involvement as co-editors in the Research Topic, and confirm the absence of any other collaboration.

## Footnotes

1 https://www.ezbiocloud.net/taxonomy

2 http://ricepedia.org/rice-as-a-plant/growth-phases
