# Supplementary figures and images for: Actinobacteria Associated With Arbuscular Mycorrhizal Funneliformis mosseae Spores, Taxonomic Characterization and Their Beneficial Traits to Plants: Evidence Obtained From Mung Bean (Vigna radiata) and Thai Jasmine Rice (Oryza sativa)
Source: Front Microbiol. 2018 Jun 11;9:1247. doi: 10.3389/fmicb.2018.01247 (PMC6004784; doi:10.3389/fmicb.2018.01247)

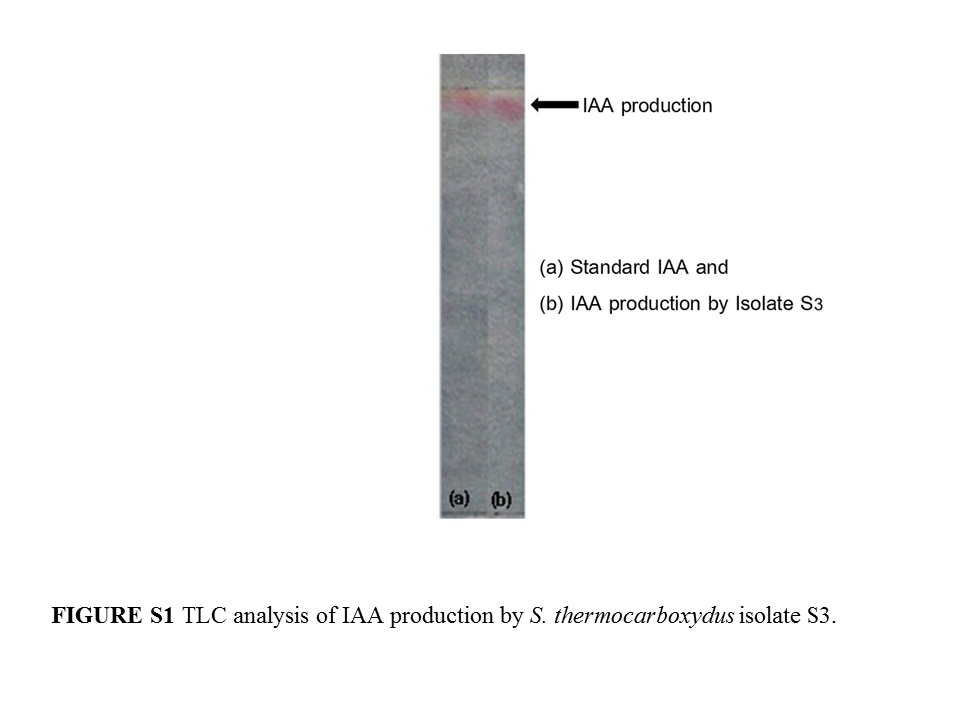

Supplement: Supplementary file 1 [file Image_1.PNG]

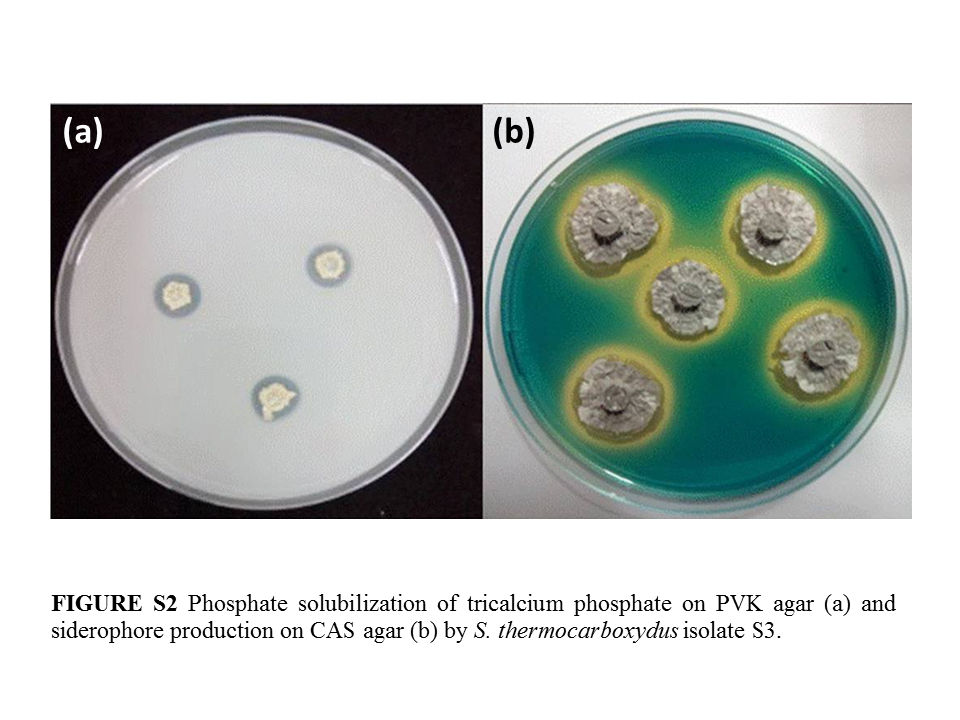

Supplement: Supplementary file 2 [file Image_2.PNG]

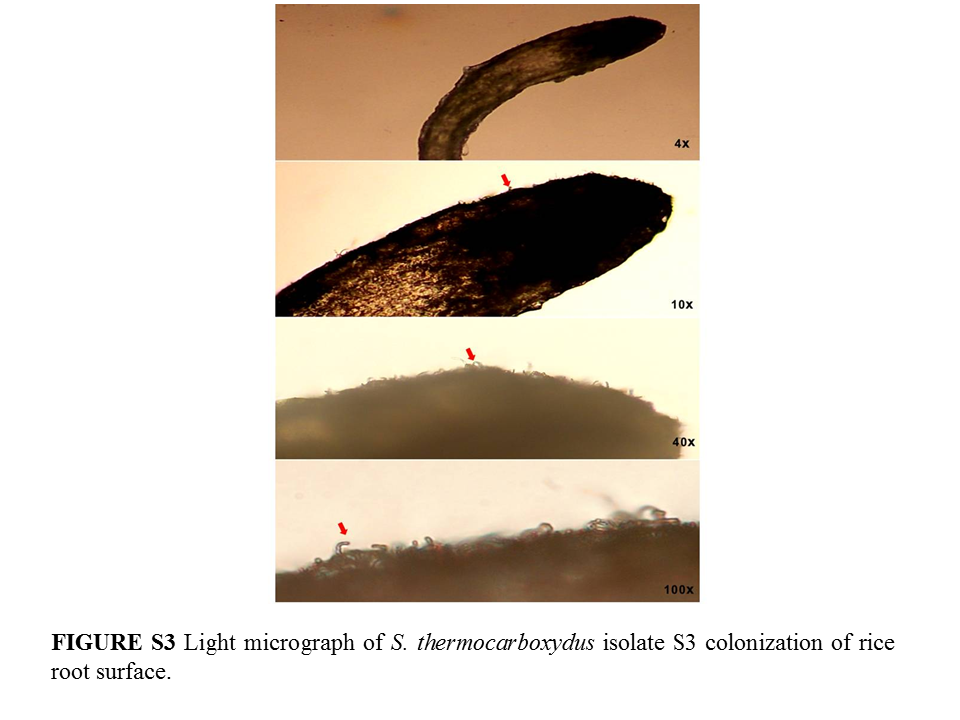

Supplement: Supplementary file 3 [file Image_3.PNG]
